# 

**Published:** 1872-03

**Authors:** 


					﻿PUBLISHED MONTHLY, AT $3 PER ANNUM, IN ADVANCE.
OQ
2
aS
*
<D
•S
'S
■5
i
P<
O
a
!S
>>
®
&
"S
’S
L
aS
CD
cd
3
p
CD
O
O
CO
m
CD
a
s
o
o
IO
J-t
CD
>
o
aS
<D
>?
aS
3
a
CD
O
rH
cn
®
1
o
o
10
>-<
<D
fl
p
I
w
o
<
EH
m
O
I*
A.TL ANTA
Nodical and Surgical Journal.
EDITED BY
J. P. LOGAN, M.D.,
AND
W. F. WESTMORELAND, M.D.,
Professor of the Principles and Practice of Surgery in Atlanta Medical College.
(Pax et Scientia, sed veritas sine timore.
VOLUME IXB-MARCH, 1872.-NUMBER 12.
ATLANTA, GEORGIA:
PLANTATION PUBLISHING COMPANY.
1872.
EACH NUMBER CONTAINS SIXTY-FOUR PAGES. ~
hl
o
tn
H
s
B
50
5
®
M
O
MO
g
£
p-
s
o
o
u
*
£
o
tn
I
cr
oc?
3:
B
at?
6
B
o
et
o'
a>
*
tr
®
P
S'
®
«-<,
o
co’
®
<T>
P-
P
et
&
o
51
o
a>
m
				

